# Strategies of the invasive tropical fire ant (*Solenopsis geminata*) to minimize inbreeding costs

**DOI:** 10.1038/s41598-019-41031-5

**Published:** 2019-03-14

**Authors:** Pauline Lenancker, Benjamin D. Hoffmann, Wee Tek Tay, Lori Lach

**Affiliations:** 1 College of Science and Engineering, James Cook University, Cairns, Qld 4878 Australia; 2 CSIRO, Tropical Ecosystems Research Centre, Darwin, NT 0822 Australia; 3 CSIRO, Black Mountain Laboratories, Canberra, ACT 2601 Australia

## Abstract

How invasive species overcome challenges associated with low genetic diversity is unclear. Invasive ant populations with low genetic diversity sometimes produce sterile diploid males, which do not contribute to colony labour or reproductive output. We investigated how inbreeding affects colony founding and potential strategies to overcome its effects in the invasive tropical fire ant, *Solenopsis geminata*. Our genetic analyses of field samples revealed that 13–100% of males per colony (n = 8 males per 10 colonies) were diploid, and that all newly mated queens (n = 40) were single-mated. Our laboratory experiment in which we assigned newly mated queens to nests consisting of 1, 2, 3, or 5 queens (n = 95 ± 9 replicates) revealed that pleometrosis (queens founding their nest together) and diploid male larvae execution can compensate for diploid male load. The proportion of diploid male producing (DMP) colonies was 22.4%, and DMP colonies produced fewer pupae and adult workers than non-DMP colonies. Pleometrosis significantly increased colony size. Queens executed their diploid male larvae in 43.5% of the DMP colonies, and we hypothesize that cannibalism benefits incipient colonies because queens can redirect nutrients to worker brood. Pleometrosis and cannibalism of diploid male larvae represent strategies through which invasive ants can successfully establish despite high inbreeding.

## Introduction

Populations arising from an introduction event often lose genetic variation because of their small founder population size^1^. Small populations are at risk of accumulating deleterious mutations via inbreeding and eventually going extinct^2,3^. However, many populations of successful invaders began as small populations having also gone through a genetic bottleneck^4^ (e.g. cheatgrass: *Bromus tectorum*^5^, Argentine ant: *Linepithema humile*^6^, house finch: *Carpodacus mexicanus*^7^, solitary sweat bee*: Lasioglossum leucozonium*^8^, Asian honey bee: *Apis cerana*^9^). This genetic paradox is well-studied in *L*. *humile* and the red imported fire ant (*Solenopsis invicta*). Both species lost genetic variation during their introductions to various areas of the globe^10,11^ and yet they are listed among the world’s most successful invaders^12^. The low genetic variability of *L*. *humile* in its invasive range may have resulted in selection for traits that encourage its spread and growth^11^. Within *L*. *humile*’s native range, workers from distinct colonies recognize and attack workers from other nests because ants from different nests have distinct cuticular hydrocarbon profiles^13^. The cuticular hydrocarbons of introduced *L*. *humile* populations became homogeneous, presumably as a consequence of genetic bottlenecks^6,13^. Therefore, the ants are unable to distinguish nest mates from outsiders and form large, dense supercolonies of interacting nests^6,13^. Supercolonies of *L*. *humile* are potentially able to direct more resources toward interspecific competition, foraging, and colony growth than colonies in their native range because of the absence of the cost of intraspecific territoriality^11^.

For other invasive Hymenoptera, such as *S*. *invicta*, genetic bottlenecks can lead to adverse consequences due to the reduction in sex-determining allele diversity in introduced populations^9,10^. The most common sex determination system of Hymenoptera is the haplodiploid sex determination system in which fertilized eggs develop into females that are diploid, and unfertilized eggs develop into males that are haploid. In some cases, the sex of the offspring is controlled by their genotype at the complementary sex determination (CSD) locus (or loci)^14^. When diploid hymenopteran individuals are homozygous at the CSD locus, they develop into sterile diploid males^15,16^. CSD and male diploidy have been found in over 60 species of Hymenoptera including *S*. *invicta*^17^.

When *S*. *invicta* queens mate only once and with a male with the same CSD genotype (i.e. matched-mating), half of their diploid offspring are homozygous at the CSD locus and develop into sterile diploid males if they are successfully reared to adulthood^18,19^. In some populations as much as 20% of *S*. *invicta* queens are match-mated and produce diploid males^18,19^. A low proportion of diploid males is fertile and their offspring are always triploid^20^. However, evidence of reproductive triploid queens has never been reported. This suggests that triploid queens are either sub-viable or executed by workers, and therefore fertile diploid males are likely a genetic dead-end^20^. Because the only function of males is reproduction, production of sterile diploid males represents an ineffective colony resource allocation and can reduce colony growth rate^10,18,21,22^.

Most of what we know about minimizing the cost of diploid male production comes from two species: *S*. *invicta* and *A*. *mellifera*. In *S*. *invicta*, polygyny (multiple-queened colonies) can help minimize the cost of sterile diploid male production induced by low genetic diversity^18^. Colonies of *S*. *invicta* can be either polygyne or monogyne (single-queened)^23–25^ but in the field diploid males are only ever found in polygyne populations^21^. Between 11.9 and 19.6% of queens from monogyne populations produce diploid males, but these queens are unable to survive under field conditions^18,21^. In the laboratory, single-queened *S*. *invicta* colonies founded by diploid male producing (DMP) queens were found to have lower growth and survival compared with non-DMP colonies because half of DMP queens’ reproductive output were males that failed to develop beyond the larval stage, instead of workers that could contribute to colony labour^18^. In the field, DMP queens that attempt to start a colony on their own would invariably fail because they would invest half of their reserves in their sterile males instead of their workers^18,21^. Presumably, only DMP *S*. *invicta* queens that are adopted into an existing polygyne colony or a queen-less monogyne colony would survive because the worker force supplied by the non-DMP queens would compensate for the production of diploid males from DMP queens^18,21,26^.

In addition to polygyny, there are three other strategies before or during colony founding that could mitigate the cost of diploid male production. Polyandry (queen mating with multiple males) could potentially reduce the occurrence of diploid male production by a match-mated queen because she could also have mated with males that do not share an allele at the CSD locus with her^9,27–29^. Execution of diploid males early in their development is another strategy to reduce the cost of diploid male production and is employed by *A*. *mellifera*^30^. However, workers of *S*. *invicta* seem unable to discriminate diploid males^18^. Finally, queens cooperatively founding a nest (pleometrosis) is another possible means of minimizing the diploid male load during colony founding because DMP queens would benefit from the worker force supplied by non-DMP queens.

The invasive tropical fire ant, *Solenopsis geminata*, is a serious agricultural and ecological pest closely related to *S*. *invicta* and has established in almost all tropical regions of the world^31^, yet little is known of its social biology. *Solenopsis geminata* originates from the Neotropics (the exact extent of its native range is unclear) and has been spread through human commerce since the 16^th^ century^31,32^. Even though diploid males have never been reported in *S*. *geminata*, its low genetic diversity across its invasive range^32^ suggests that *S*. *geminata* potentially went through a genetic bottleneck and that diploid males are probably common. Both social forms, monogyne and polygyne, have been reported to occur in native populations of *S*. *geminata*^24^ and invasive populations in Florida^23,33^ and the Galapagos islands^34^. Colonies have been found to be exclusively monogyne in Taiwan^35^. Its social form remains unknown for the rest of its invasive range.

Initial observations of the Australian *S*. *geminata* population we used in our study indicated that newly mated queens dispersed via mating flights and were capable of independent founding when placed under laboratory or controlled field conditions. Independent founding is more frequent in monogyne ant populations but, in some species, queens from polygyne populations found colonies independently^36^. In some ant species, queens originating from polygynous colonies can found a colony under laboratory conditions in the absence of workers and other queens, demonstrating that the founding mode of polygynous ants may be more diverse than thought (e.g. *Solenopsis invicta*^37^, *Anoplolepis gracilipes*^38^, *Pachycondyla villosa*^39^ and, *Lasius neglectus*^40^). We also observed in our *S*. *geminata* population that founding queens sometimes produced unusually large larvae, which in *S*. *invicta* typically indicates diploid male production^21^. Queens of *S*. *geminata* found colonies claustrally, whereby the queen seals herself in a chamber and rears her first brood using her fat reserves^41^. When the first workers emerge, they leave the nest to forage and feed the queen and her brood. Rapid growth is essential for the survival of claustrally founded colonies^18^. Therefore, diploid male production would likely be highly detrimental for these colonies’ growth and survival. Whether *S*. *geminata* produces diploid males or has strategies that would minimize the costs of diploid male production and increase colony founding success has not previously been studied.

Our work aimed to establish whether *S*. *geminata* queens produced diploid males, how common diploid male production is, and whether colony founding strategies can compensate for the genetic load associated with diploid male production. We used a combination of microsatellite analyses on field-collected *S*. *geminata* males and queens and a laboratory experiment in which we simulated a claustral founding scenario with haplometrosis and pleometrosis treatments to: (1) determine whether diploid males exist, and their prevalence, (2) estimate the number of males queens mate with, (3) determine whether diploid male production hinders colony growth and, (4) investigate whether strategies such as pleometrosis or brood cannibalism alleviate the potential burden of rearing sterile males. Our findings will improve our understanding of one of the most fundamental questions in invasion biology research: how invasive species overcome low genetic diversity and inbreeding.

## Material and Methods

### Queen and male collection

We collected newly mated queens of *S*. *geminata* after their nuptial flight, between March and April 2015 and 2016 at Humpty Doo (Supplementary Fig. A1), Northern Territory, Australia (−12.5722°, 131.0842°), as they flew to a veranda light and dropped to the ground to found a nest in the early evening. We used some of these queens for our colony founding experiment (see colony founding experiment section) and the remainder for spermathecal dissections (see spermatheca dissections section).

To determine the presence of diploid males in the field we collected eight adult males from each of ten nests at six sites in the Northern Territory (Supplementary Table A1 and Fig. A1) between January and March 2014. The males were placed in 99% ethanol at −18 °C until DNA extraction (see DNA extraction and microsatellite genotyping section).

### Spermatheca dissection

We isolated sperm from 40 mated queens collected in 2015 following the method of Tay & Crozier (2001)^42^. Each sperm sample was stored in 10 µL isotonic buffer (1.76 mM NaCl) at −18 °C until DNA extraction. The head and thorax of individual dissected queens were stored in 99% ethanol at −18 °C until DNA extraction (see DNA extraction and microsatellite genotyping section).

### Colony founding experiment

The 1187 queens collected in 2016 yielded 115 ± 12 replicates per treatment. The queens were weighed and established in trials of 1, 2, 3 or 5 queens per colony within 15 to 40 hours after collection. Nests consisted of a 15-mL centrifuge tube, half filled with water retained by a cotton plug. Queens were retained by a second cotton plug inserted in the tube mouth. We inserted the nesting tubes into a polystyrene sheet to keep the nesting chambers in the dark. We allowed queens to initiate nests for 23 days, which was 1 day after the emergence of the first generation of workers in our pilot study. Because *S*. *geminata* queens are capable of founding their colonies claustrally^43^, they did not need to be fed during the experiment.

We recorded queen mortality and the cause of death three times per week. We considered dismembered queens to have been executed by other queens and dead intact queens to have died of natural causes. We also recorded the presence of distinctly large larvae and their position within the nest three times per week. On the 24^th^ day we terminated the colonies by freezing, weighed each queen and all brood, and counted the number of eggs, larvae, worker pupae, and adult workers of the colonies in which all the queens survived (n = 380, 95 ± 9 replicate per treatment). Unusually large larvae distinguishable from the second instar larval stage were counted and weighed separately from the rest of the brood. As detailed in the Discussion, we considered large larvae to be males and not queens because investment in queen production is very unlikely during the colony founding stage^25,43^. We opportunistically selected 15 large larvae, out of the 109 we observed, for ploidy determination using microsatellite markers to determine whether they were haploid or diploid male larvae (see DNA extraction and microsatellite genotyping section). We left the other larvae in their nest to observe how the queens were treating them.

### DNA extraction and microsatellite genotyping

We extracted DNA from 80 adult males collected from ten field colonies (8 males per colony), 40 sperm samples from mated queens immediately following their nuptial flights, and 15 large larvae from the colony founding experiment. We also extracted DNA from the 40 queens that were dissected for sperm sampling, to enable distinction of multiple mating from potential maternal contamination. For larvae, males, and queens we used the Zymo Research Tissue and Insect DNA MiniPrep^TM^ kit following the supplier’s instructions. For the sperm, we used the Qiagen DNeasy Blood and Tissue kit and the Zymo Research DNA Clean and Concentrator^TM^ following the supplier’s instructions.

Based on the criterion of maximal allele detection^32^, we chose six microsatellite markers (Supplementary Table B1) for the adult males and three markers for the queen, sperm, and larvae. PCR conditions are described in Appendix B (Supplementary Information). PCR for the queen, sperm and larvae were multiplexed and submitted to the Australian Cancer Research Foundation Biomolecular Resource Facility at the John Curtin School of Medical Research, Australian National University, for genotyping. We used the proprietary software ‘Geneious®’ (Biomatters Ltd., Auckland, New Zealand) to visualize trace files, fit the internal ladder, and identify microsatellite alleles. The highest peaks within the allele size range for *S*. *geminata* (Supplementary Table B1) were determined. Heterozygous male larvae were scored as diploid. We inferred the queen’s mating frequencies by scoring the alleles for the sperm and compared queen and sperm genotypes for contamination.

### Statistical analysis

We analysed the colony founding experiment data using R version 3.3.2^44^ and functions from the stats package (R Development Core Team 2009) unless otherwise mentioned. We used a generalized linear model (GLM, glm function) followed by ANOVA F-test (Anova function with test = F in the car package^45^) with the total number of brood and adult workers per colony as the response variable, the number of queens per colony (1, 2, 3 or 5), the presence of large larvae, and the mean initial queen weight as fixed factors. We used count rather than weight data as the response variable for our models because the two were highly correlated (Pearson’s correlation, r = 0.82, t = 27.516, df = 378, P < 0.0001) and accurate measurement of brood weight can be difficult to achieve considering the size of the eggs and ease of damaging larvae. We used a quasi-Poisson error structure to account for overdispersion^46,47^.

To test the effect of large larvae production on egg, regular-sized larva, and the sum of worker pupae and adult worker production, we used three generalized linear models (glm function) with a quasi-Poisson error structure followed by ANOVA F-test with the response variable being either the number of eggs, number of larvae, or sum of worker pupae and adult workers per colony and with explanatory variables being the number of queens per colony (1, 2, 3 or 5), the presence of large larvae, and the mean initial queen weight. To test whether large larvae production and pleometrosis influenced the average weight loss of founding queens during the experiment, we used a linear model (lm function) with average queen weight loss as the response variable and the number of queens per colony and the presence of large larvae as explanatory variables. To test whether the number of brood and adult workers produced per queen (sum of brood and adult worker count divided by the number of queens) varied with queen treatment, we used a linear model (lm function) with the number of brood and adult workers produced per queen as the response variable and the number of queens per colony and the presence of large larvae as explanatory variables. We compared the equivalent distribution of average queen weight in each treatment (i.e. number of queens per colony) with a Kruskal-Wallis test (kruskal.test function). We verified whether queen execution was higher in colonies with large larvae than in colonies without using a contingency chi-square test (chisq.test function).

We conducted additional analysis on the haplometrosis (single queen) treatment. We analysed the effect of the initial queen weight on the brood and adult worker count with a Wilcoxon signed-rank test (wilcox.test function). We tested whether weight loss differed between queens that did and did not have brood at the end of the experiment using Wilcoxon rank sum test (wilcox.test function). We tested whether queens that died of natural death were lighter than surviving queens using a Kruskal-Wallis test (kruskal.test function).

For all models, we plotted the residuals to check for their homoscedasticity, independence, and normality (plotresid function in the RVAideMemoire package^48^) and, where appropriate, we used post hoc Tukey tests to make pairwise comparisons (lsmeans function in the lsmeans package^49^) which is the best available method when sample sizes are unequal^50^.

## Results

### Diploid males in field colonies

Diploid males were found in 8 of the 10 field colonies (Supplementary Table A2). According to microsatellite allele scoring, from one to all (13–100%) of the 8 sampled adult males per colony were diploid. Of the six microsatellite DNA markers that we used, two markers (i.e., Ms16C121 and Ms33Sol11) were sufficiently polymorphic to detect heterozygosity. Out of the 80 male samples, we successfully amplified 69 male DNA samples at both these loci, and 8 at one locus while amplification failed at both loci for 3 samples. Heterozygosity at one locus indicates diploidy. In total, we found that 31 males (44.9%) were heterozygous, 26 at one locus and 5 at two loci. Five field colonies had more than two alleles at a given locus: H2 at 2 loci (Ms16C121 and Ms14C334), V1, E1 and, S1 at one locus (Ms33Sol11 for V1 and E1 and, Ms14C334 for S1) while the remaining five had two alleles per locus at most.

### Queen mating frequency

We found all 40 queens to be monandrous (i.e. one allele per individual sperm sample), so there was no need to cross examine sperm DNA with maternal DNA to check for maternal contamination. We successfully amplified 31 of 40 sperm DNA samples for all three microsatellite loci, while the remaining 9 samples amplified successfully for two loci. We successfully amplified 15 queen DNA samples for all three microsatellite loci, 9 DNA samples for two loci, and 12 DNA samples for one locus. None of the queens were triploid. All sperm DNA samples had one allele at all loci or the locus for which they were successfully amplified. We used the sperm genotype frequency from 32 males (Table D2) which were successfully genotyped at all three loci to calculate the probability of n = 1–32 queens having mated with two males sharing the same genotype (see Appendix D for more details on the calculation). We found that the probability of missing one double-mated queen (i.e. double-mating frequency: 3.1%) was 0.121 (Fig. D1). The probability of having missed double mating in 12.5% of the population and above was negligible (from 2.14 × 10^−4^ to 4.46 × 10^−30^). Therefore, polyandry is unlikely in our population but could occur at low frequency.

### Colony founding experiment

Of the 487 colonies reared (n = 122 ± 6 per treatment), 106 had queens that died before the end of the experiment. Of these, we deemed 67 colonies had queens die naturally, 28 had signs of queen execution, and 11 colonies had both. The frequency of queen mortality was too low to compare it across treatments. Queens were executed from day 2 to day 23 (mean ± SD: 13.2 ± 6.0 days) of the experiment. In the single queen treatment, the initial weight of queens that died naturally was the same as surviving queens (Kruskal-Wallis test, Kruskal-Wallis χ^2^ = 29.601, df = 38, P = 0.833).

We observed a total of 109 large larvae in 85 out of 380 colonies. None of these larvae developed into pupae before we terminated the colonies. Of the 15 large larvae we had selected for ploidy determination, 10 were successfully amplified at two to three markers. Three of these larvae were heterozygous at two markers and four at one marker and were scored as diploid. We detected one allele per marker for the remaining three larvae. We observed that 40% of the diploid male producing colonies had placed their large larvae in the refuse pile with the colony waste, away from the rest of the brood. We did not observe large larvae to be tended by queens, except on two occasions in which we observed queens in two different colonies tapping a larva placed in their respective refuse piles with their antennae. Following the results from our microsatellite DNA analyses, common behaviour displayed by queens towards large larvae and, additional evidence detailed in the Discussion, we considered all large larvae to be diploid males in the subsequent analyses. We hereafter refer to the queens and colonies that produced these large larvae as diploid male producing (DMP).

**Figure 1:**
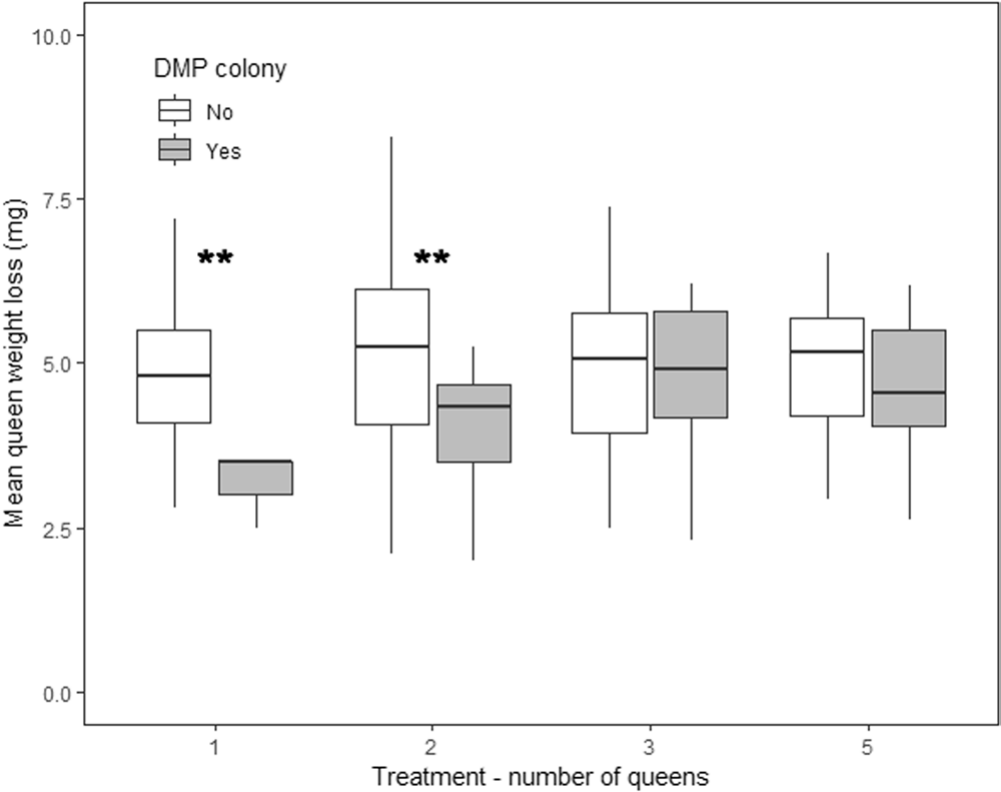
Mean queen loss of weight between the start and the end of the experiment for each queen treatment separated between DMP (diploid male producing) colonies and non-DMP colonies. The interaction between diploid male production and the number of queens had a significant effect on the mean queen weight loss (LM: ANOVA, χ^2^ = 11.7166, P < 0.01). **Indicates a significant difference between DMP colony and non-DMP colonies for the corresponding queen treatment (post hoc tests P < 0.01). One queen: non-DMP n = 97, DMP n = 5, two queens: non-DMP n = 79, DMP n = 16, three queens: non-DMP n = 68, DMP n = 29, five queens: non-DMP n = 51, DMP n = 35.

We found evidence of queens executing their diploid male larvae. We found that 73.4% (n = 80/109) of diploid larvae disappeared between days 2 and 12 (mean ± SD: 3.8 ± 1.8 days) after we had observed them. Large larvae disappeared in 43.5% of DMP colonies. There were too few occurrences to compare the number of executed diploid male larvae among treatments (Supplementary Fig. C3). Queens were the only adult ants in our colonies, which suggests that the queens cannibalized their diploid male larvae or fed them to other larvae. Queens from single and two-queened DMP colonies had lost less weight on average than non-DMP colonies but there was no difference in queen weight loss for three and five-queened colonies (Fig. 1; LM: ANOVA, χ^2^ = 11.7166, P < 0.01; post hoc tests P < 0.01 for pairwise comparisons DMP vs. non DMP in single queen and two-queened colonies, P > 0.05 for all other pairwise comparisons). In 34.1% of DMP colonies (n = 29/85), diploid male larvae were first found with the colony refuse before disappearing. We thought diploid male larvae could have died before being placed in the refuse pile by the queens. However, discarded larvae looked live and intact even after remaining in the refuse pile for several days, and we confirmed with a microscope that two of the discarded diploid male larvae were alive.

We estimated the proportion of DMP queens to range from 4.9% to 10.0%. We found that 4.9% of colonies with single queens produced diploid males, that would normally give us the proportion of DMP queens in the population. However, this number is likely an underestimate because 29.4% of queens in the single queen treatment did not produce any brood, and without any larvae it was not possible to differentiate non-DMP queens from DMP queens. If we take out queens that did not produce brood, 7.5% of single queens were DMP. We found that 16.8%, 29.9%, and 45.3% of 2-, 3- and 5-queen colonies, respectively, produced diploid males. To obtain another estimation of DMP queen frequency, we divided the frequency of DMP colonies by the number of queens for each multiple queen treatment. We obtained a DMP queen proportion of 8.4% for 2-queen colonies, 10.0% for 3-queen colonies and 9.1% for 5-queen colonies. We found that queen execution occurred in 10.9% of the pleometrotic colonies and that queen execution was not higher in DMP colonies than in non-DMP colonies (contingency chi-square test, χ^2^ = 0.25721, df = 1, P = 0.612).

**Figure 2:**
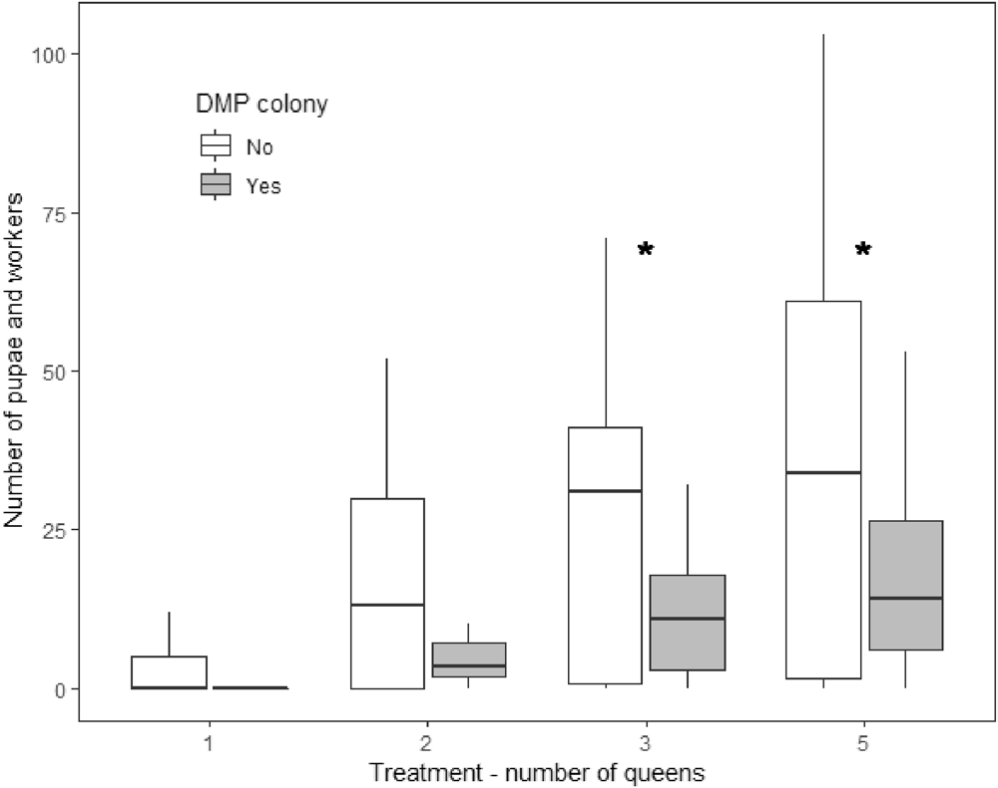
Number of worker pupae and adult workers produced for each queen treatment separated between DMP (diploid male producing) colonies and non-DMP colonies. DMP colonies had significantly fewer worker pupae and adult workers than non-DMP colonies (Table 1); ^*^ Indicates a significant difference between DMP colony and non-DMP colonies for the corresponding queen treatment (post hoc tests P < 0.05). One queen: non-DMP n = 97, DMP n = 5, two queens: non-DMP n = 79, DMP n = 16, three queens: non-DMP n = 68, DMP n = 29, five queens: non-DMP n = 51, DMP n = 35.

The production of diploid males led to a reduction in the production of pupae and adult workers in DMP colonies which pleometrosis could alleviate. Our GLM with the number of worker pupae and adult workers as the response variable showed that DMP colonies had significantly fewer worker pupae and adult workers than non-DMP colonies (Fig. 2; Table 1; post hoc tests P < 0.05 for pairwise comparisons DMP vs. non DMP in three-queened and five-queened colonies, P > 0.05 for all other pairwise comparisons). Increasing the number of founding queens in DMP colonies increased the number of brood and adult workers at the end of the experiment (Supplementary Fig. C1; post hoc tests DMP colonies P < 0.001 for pairwise comparisons two vs five queens, P < 0.01 for one vs five and three vs five queens, P < 0.05 for two vs three queens, P > 0.05 for all other pairwise comparisons). There was no difference in the combination of brood and adult worker counts between DMP colonies and non-DMP colonies by treatment (Table 1 and Supplementary Fig. C1). We were able to distinguish worker from diploid male larvae from the second instar larval stage and excluded diploid male larvae we had identified from the brood count. We found similar results when analysing eggs and worker larvae separately (Table 1).

**Table 1:** Summary of generalized linear model results for each response variable in the colony founding experiment. ‘x’ represents the interaction terms.

Variables	*df*	F		*p*
Number of worker brood and adult workers	quasi-poisson, n = 380			
Number of queens per colony	3	141.52		<2.2e-16
Presence of diploid male larvae	1	3.60		0.059
Queens x diploid male larvae	3	0.57		0.63
Mean initial queen weight	1	2.55		0.11
Number of eggs	quasi-poisson, n = 380			
Number of queens per colony	3	108.63		<2.2e-16
Presence of diploid male larvae	1	0.30		0.59
Queen x diploid male larvae	3	0.64		0.59
Mean initial queen weight	1	0.52		0.47
Number of larvae	quasi-poisson, n = 380			
Number of queens per colony	3	46.68		<2.2e-16
Presence of diploid male larvae	1	0.29		0.59
Queen x diploid male larvae	3	0.39		0.76
Mean initial queen weight	1	4.02		0.04
Number of worker pupae and adult workers	quasi-poisson, n = 380			
Number of queens per colony	3	54.89		<2.2e-16
Presence of diploid male larvae	1	34.76		<0.01
Queen x diploid male larvae	3	1.11		0.35
Mean initial queen weight	1	2.65		0.10

The more queens founding a colony together, the more brood the colony had at the end of our experiment. Colonies with more queens had significantly more brood and adult workers (Table 1; Tukey post hoc tests P < 0.01 for all pairwise comparisons) but the number of brood and adult workers per queen did not vary with the number of queens per colony (LM: ANOVA, χ^2^ = 36.038, P < 0.01; post hoc tests P > 0.05 for all pairwise comparisons, Supplementary Fig. C2). Colonies with more queens had more eggs (Table 1; Tukey post hoc tests P < 0.01 for all pairwise comparisons) and more larvae (Table 1; Tukey post hoc tests P < 0.01 for all pairwise comparisons except for 2 queens vs. 1 queen and 2 queens vs. 3 queens for which P > 0.05). We found no live brood in 29.4% of colonies in the single queen treatment and 1.8% of colonies in the multiple queen treatments. All of these colonies had eggs that were dead at the end of the experiment, and their nests were full of mould.

The initial weight of queens influenced some of the brood production. The weight distribution of newly mated queens before the start of the experiment (n = 1,013) was unimodal (Supplementary Fig. C4). Most queens (99%) weighed between 11 and 16 mg while a few (1%) appeared to be outliers, weighing less than 11 mg. We did not notice lighter queens to be unusually smaller (microgynes) than heavier queens. The initial mean queen weight did not differ among treatments (Kruskal-Wallis test, Kruskal-Wallis χ^2^ = 2.7102, df = 3, P = 0.439). The total number of brood and adult workers produced by each colony did not change with the average queen initial weight in pleometrotic treatments (Table 1). But in the single queen treatment, queens that were heavier at the start of the experiment had more brood and adult workers than lighter queens (Wilcoxon signed-rank test, V = 5253, P < 0.0001). Queens that produced brood lost more weight than brood-less queens. Haplometrotic queens that had brood at the end of the experiment lost 39 ± 8.5% (mean ± SD) of their initial weight whereas queens that did not have brood lost 36 ± 5.1% (Wilcoxon rank sum test, W = 789, P < 0.05). Regardless of treatments, colonies with heavier initial average queen weight had significantly more larvae than colonies with lighter average queen weight, whereas average initial queen weight had no significant effect on number of eggs and the sum of worker pupae and adult workers (Table 1). The number of queens per colony did not affect the average queen weight loss (LM: ANOVA, χ^2^ = 11.7166, P > 0.05).

## Discussion

Genetic bottlenecks can cause populations to accumulate deleterious mutations and be at risk of extinction^2,3^. However, many invasive species successfully establish despite having experienced a genetic bottleneck^4,6,32^. Understanding how invasive populations with low genetic diversity become successful invaders is one of the central questions of invasion biology. As expected given the low genetic diversity within our *S*. *geminata* population, we found that diploid males were common in the field (n = 8/10 colonies) and that their frequency within colonies varied greatly (13–100% of the sampled adult males). Diploid males may be more common than we estimated. We found that 47.3% of queens (n = 36) were homozygous. If the frequency of homozygosity is similar between queens and males, 47.3% of males scored as haploid may have been homozygous diploid males. Therefore, diploid male frequency could range from 54 to 100% instead of 13–100% of males sampled per colony. To our knowledge, this is the first evidence of diploid male production in *S*. *geminata*. We also found that *S*. *geminata* can use two strategies to minimize the cost of diploid male production: pleometrosis and execution of diploid male larvae. Polyandry could have potentially reduced the occurrence of DMP queens but, we found that the queens were always single-mated.

Our results confirmed that the genetic diversity of *S*. *geminata* in Australia was low, presumably because this population reflected the expected genetic bottleneck during the spread of *S. geminata* through the Indo-Pacific region^32^. For example, we found that the three microsatellite DNA markers used on queen samples had 3 to 7 alleles per locus (Supplementary Table D1) whereas the same markers used in a worldwide study of *S*. *invicta* had 13 to 22 alleles per locus in some sites situated within the native range of *S*. *invicta*^51^. This genetic bottleneck most likely contributed to the production of diploid males. We estimated the frequency of DMP queens in our lab colonies to be between 4.9% and 10.0% which is lower than the newly mated DMP queen frequency of *S*. *invicta* originating from monogyne (11.9 to 19.6%^18,21^) and polygyne populations (13.9%^21^) in its invasive range. It is possible that triploid queens may be present in small numbers among the Australian population of *S*. *geminata*, but we failed to detect these queens among newly mated queens that were collected for this study. If triploid queens are absent in the field, it may indicate that diploid males are aspermic, contribute to the production of sub-viable queens, or that reproductive triploid queens are executed by workers as suggested for *S*. *invicta*^15^. The low genetic diversity in our population made diploidy difficult to detect in our large larvae. In *S*. *invicta*, the presence of large larvae with the worker brood of founding queens typically indicates diploid male production^21^. Sexual larvae of *S*. *geminata* are distinctly larger than worker larvae as with *S*. *invicta*^25^, and therefore, theoretically, a large larva could be either a male larva or a queen larva. However, it is highly unlikely that incipient colonies would invest in queen production^25,43^. Any large larvae would therefore be the result of either unmated queens that are only able to produce haploid males or match-mated queens producing diploid males. We dissected 101 newly mated queens’ spermathecae as part of this study and found all to have mated. The lines of evidence taken as a whole suggest that these large larvae are diploid males which queens from our *S*. *geminata* population commonly produce because of the population’s small genetic diversity.

The production of diploid males imposes a cost to the colony during the founding phase according to the results from our colony founding experiment. The production of worker pupae and adult workers was lower in DMP colonies. This result is consistent with the idea that DMP colonies were unable to successfully rear as much worker brood to a late developmental stage as non-DMP colonies because the queen’s body reserves were being depleted by rearing costly diploid male larvae. In the related *S*. *invicta*, the production of sterile diploid males also represents a burden to the colony^10,18,21,22^. However, we did not find deleterious effects of diploid male production on the total brood and adult workers produced. Extension of the colony founding experimental time frame, such as past the claustral period, may be needed to detect more pronounced effects of diploid male production on colony founding. We focused on the claustral phase so that we could draw conclusions about founding queen strategies without having to control for nascent worker foraging abilities.

We also found that pleometrosis was an efficient strategy to minimize the cost of diploid production and provided a distinct advantage to our colonies compared to haplometrosis. For example, three and five-queened colonies were 45.1–49.6% more likely to have worker pupae at the end of the claustral period and reared on average 5 to 7 times more pupae than haplometrotic colonies. The worker force supplied by DMP queens’ nestmates increases their chances of successful colony founding. Queens in the multiple queen treatments were also more likely to survive the claustral phase than queens in the single queen treatment. Most ant species are exclusively haplometrotic but, some species can found colonies using either mode of colony founding^52^. Queens of *S*. *invicta* in their invasive range are more likely to join each other during colony founding when local queen density is high^41^. Queens founding a colony cooperatively are more successful during the claustral and incipient phase than single-founding queens, and pleometrosis increases brood production and queen survival for several ant species (e.g. *S*. *invicta*^53^, *Iridomyrmex purpureus*^54^, *Myrmecocystus mimicus*^55^, *Pogonomyrmex californicus*^56^). Pleometrotic colonies can also begin foraging earlier than single-queen colonies (e.g. *Veromessor pergandei*^57^), and the benefit from the initial boost in brood production can remain long after the end of the claustral period (e.g. *M*. *mimicus*^55^). Because pleometrosis is more common in areas with high ant density, and cooperative founding increases colony establishment success^58^, we would expect pleometrosis to be more common in successful invasive ant taxa. Pleometrosis would also especially benefit species with a high prevalence of DMP queens, but these hypotheses have not been investigated to the best of our knowledge.

Our findings about the benefits of pleometrosis are unlikely to be an artefact of a laboratory experiment. Combined results from our laboratory experiment and field obtained data indicate support for independent and pleometrotic colony founding in the field resulting in polygynous colonies. Four lines of evidence support independent pleometrotic founding in the field. First, field observations of our population indicate that queens disperse via mating flights. Second, most of our queens successfully founded a colony independently with or without other queens. For example, a large majority (68.1%) of our colonies successfully reared brood to the pupal stage. Third, queen execution was rare (10.9%) with most of the queens readily accepting each other and rearing their brood together. Finally, pleometrosis provided a clear advantage to the founding colonies and minimized the effect of diploid male production. We also have several lines of evidence in favour of polygyny in our population. First, five of our ten field colonies for which we genotyped males had more than two alleles per locus. Workers in the *Solenopsis* genus are unable to produce males because they do not have ovaries^59^. Therefore, these five colonies probably contain more than one queen. The remaining colonies had between one and two alleles per locus, probably due to the low diversity at our six loci (we detected between two and seven alleles at each locus). Second, the weight distribution of queens we used in the colony founding experiment was unimodal (Supplementary Fig. C4); in Florida, *S*. *geminata* queens originating from monogynous colonies are dimorphic, and the smaller queens are incapable of independent founding whereas the weight distribution of polygynous queens is unimodal^43^. Third, the presence of diploid males in the field may indicate polygyny as has been observed in the closely related *S*. *invicta*. In *S*. *invicta*, diploid males are only ever found in polygyne populations and are absent from monogyne populations in the field, although newly mated queens from monogyne populations produce diploid males under laboratory conditions^18,21^. Our field and laboratory-based evidence suggest that the northern Australian *S*. *geminata* population is polygynous and its queens found new colonies independently. Independent founding is more frequent in monogyne ant populations but, in some species, queens from polygyne populations found colonies independently^36^. Several species with polygyne colonies are also capable of independent founding under laboratory conditions, which suggests that colony founding strategies might be more complex than previously suggested^37–40^.

Selective execution of diploid male larvae appears to be a second strategy employed by *S*. *geminata* to lessen the cost of inbreeding. We found evidence in 43.5% of our DMP colonies that queens of *S*. *geminata* selectively cannibalize diploid male larvae or feed them to worker larvae. This behaviour may benefit DMP queens because it could prevent further investment in costly genetic dead-ends and allows queens to reclaim nutrients that they can redirect towards worker brood. The reclaiming of resources may explain why diploid male production did not increase queen mortality and why queens in single and two-queened DMP colonies lost less weight than non-DMP colonies. We acknowledge, however, that the low number of DMP colony replicates for these treatments (n = 5 and n = 16, for one and two-queened colonies, respectively) might have resulted in the significantly higher weight loss in non-DMP colonies. However, because we would expect that queens in DMP colonies would lose more weight than those in non-DMP colonies, even a finding of no significant difference, as we observed in the three and five-queened colonies, provides support for our hypothesis.

Larval execution is common in lab-reared *S*. *invicta* colonies with more than five queens^53^, but *S*. *invicta* workers have not been found to discriminate diploid male larvae from worker brood for execution^18^. In fact, workers cannot differentiate diploid from haploid males at the adult stage either, even if diploid males are slightly larger than their haploid counterparts^18,21^. That we found adult diploid males in our field survey indicates that workers rear diploid males and are probably unable to differentiate them from haploid males. Other Hymenoptera, such as *A*. *mellifera* workers, can recognize diploid drones hours after hatching and cannibalize them^30^. *Monomorium pharaonis* workers can differentiate sexual from worker larvae and, when queens are present, workers selectively cannibalize sexual larvae^60^. Workers of *Formica exsecta* eliminate male larvae before the pupal stage, probably as a response to resource limitation^61^. Some of our queens selectively eliminated their diploid male brood, and therefore *S*. *geminata* queens may be able to differentiate diploid male larvae from worker larvae based on size but, whether queens can differentiate diploid from haploid male larvae remains unknown.

Finally, although polyandry could reduce the occurrence of diploid male production and help overcome the cost of inbreeding, we found that this strategy was unlikely to occur in our population. Polyandry can potentially reduce the occurrence of match mating and increase the number of alleles at the CSD locus (or loci) carried by mated queens forming a founder group^9,27–29^. All 40 sperm DNA samples we collected from queen spermatheca had one allele at all loci or the single locus for which they were successfully amplified. The probability of one queen having mated with two males sharing the same genotype, and therefore not being detected as having double-mated, was 0.121 for our samples. The probability that we failed to detect double mating drops rapidly. For example, the probability of having missed 12.5% of double mating is 2.14 × 10^−4^. Therefore, most queens of *S*. *geminata* are likely to be single-mated in northern Australia. *Solenopsis geminata* queens were also found to be monandrous in Florida^62^, which indicates that single mating might be the norm for this species. However, polyandry could potentially be more common in some populations. *Solenopsis invicta* queens were considered to be exclusively monandrous^22^ until Lawson and colleagues (2012)^63^ found that up to 20% of the queens are polyandrous in some populations.

Invasive species must overcome challenges linked to inbreeding to successfully establish and then maintain viable populations^1^. Pleometrosis and diploid male larvae execution are two strategies *S*. *geminata* queens can use to successfully establish new colonies despite high inbreeding that causes some queens to have half of their workers develop into sterile males. It would be useful to know whether queens can differentiate diploid from haploid male larvae, what type of recognition cue(s) they use, and whether this behaviour is restricted to the claustral period. It would also be illuminating to test whether selective larval execution only occurs in invasive populations of *S*. *geminata* as a response to the genetic load of sterile diploid males.

## Supplementary information

Supplementary information

## Data Availability

The article’s supporting data has been archived in the Research Data (Tropical Data Hub) repository at James Cook University, Australia (10.25903/5b4fd2f52dba9).
