# Supplementary material for: Strategies of the invasive tropical fire ant (Solenopsis geminata) to minimize inbreeding costs
Source: Sci Rep. 2019 Mar 14;9:4566. doi: 10.1038/s41598-019-41031-5 (PMC6418234; doi:10.1038/s41598-019-41031-5)
Supplement: Supplementary file 1 — Supplementary information [file 41598_2019_41031_MOESM1_ESM.pdf]

SUPPLEMENTARY INFORMATION

Strategies of the invasive tropical fire ant (*Solenopsis geminata*) to minimize inbreeding costs

Authors: Pauline Lenancker[*1,2], Benjamin D. Hoffmann[2], Wee Tek Tay[3] and Lori Lach[1]

***** Corresponding author: Pauline.Lenancker@my.jcu.edu.au

[1] College of Science and Engineering, James Cook University, Cairns, Qld 4878, Australia

[2] CSIRO, Tropical Ecosystems Research Centre, Darwin, NT 0822, Australia.

[3] CSIRO, Black Mountain Laboratories, Canberra, ACT 2601, Australia

Appendix A. Collection sites and ploidy results of field-sampled males.

Table A1. Male collection sites.

Colony code	Site	Collection date	GPS coordinates	
			Latitude	Longitude
D1	Douglas-Daly	29-Jan-14	-13.830017	131.226036
D2	Douglas-Daly	29-Jan-14	-13.834688	131.184547
H1	Humpty Doo	25-Jan-14	-12.548661	131.036591
H2	Humpty Doo	25-Jan-14	-12.548661	131.036591
H3	Humpty Doo	25-Jan-14	-12.548661	131.036591
E1	East Point	15-Feb-14	-12.412442	130.82912
P1	Pirlangimpi	01-Mar-14	-11.573308	130.581122
S1	Snake Bay	01-Mar-14	-11.42062	130.661118
V1	Virginia	25-Jan-14	-12.548661	131.036591
V2	Virginia	15-Feb-14	-12.547171	131.036639

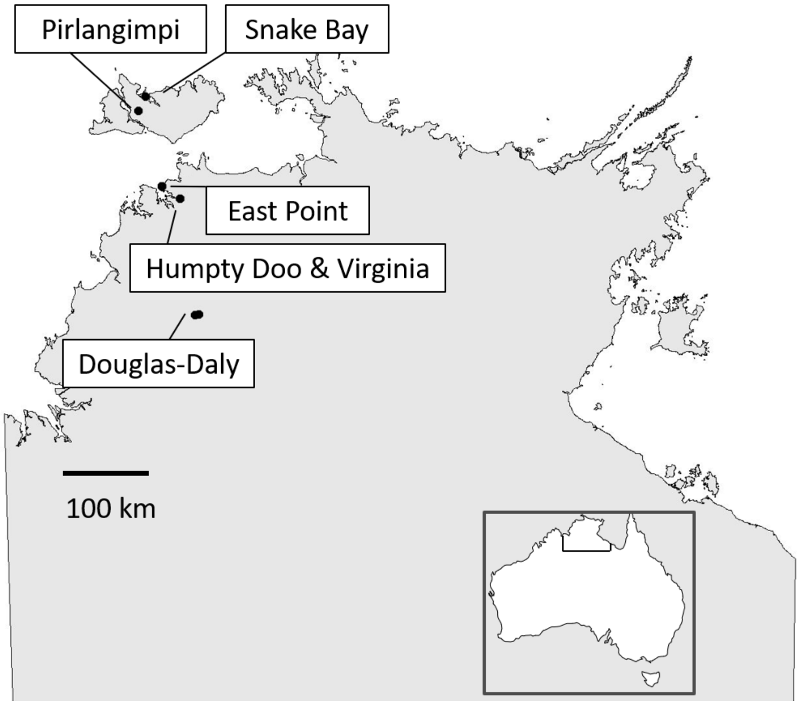

Figure A1. Map of the male collection sites in the Northern Territory, Australia.

Table A2. Number of heterozygous males scored as diploid for each field-collected colony.

Colony	D1	D2	H1	H2	H3	E1	P1	S1	V1	V2
1 marker	4	4	0	2	2	2	7	0	1	4
2 markers	1	4	0	0	0	0	0	0	0	0

Note: Total males sampled n = 80, successful amplification for all six microsatellite loci n = 48, five loci n = 23, four loci n = 1, three loci n = 1, and one locus n = 5 samples, failed amplification at all loci n = 2.

Appendix B. PCR protocol for queen, larva and male DNA and microsatellite primer sequences

All DNA fragments were amplified using PCR based on the methods of Tay & Crozier (2000), modified and optimized for *S. geminata* microsatellite markers. Each amplification (in a final 10µL reaction volume) required 1.5 µL of DNA (undiluted for queens, larvae and males, 1:10 diluted for sperm), 0.2mM dNTPs, 0.2µM of each forward primer and reverse primer, 0.5 unit of Phusion® Taq DNA polymerase (5u/µL), 1mM $MgCl_2$, and 1x Phusion® Buffer. The PCR conditions were as follows: initial denaturation step at 95˚C for 2.5 minutes; 37 cycles comprising 30 seconds of DNA denaturing at 95˚C, specific primer annealing temperature (Table A1) for 30 seconds, 30 seconds DNA extension at 72˚C; and a final extension step of 5 minutes at 70˚C. Post PCR incubation was at 10˚C. For each individual sample, we multiplexed and submitted for genotyping 2.5 µL of each of the PCR amplicons for Ms19c367, Ms14c334 and Ms4Sol55 and, 2 µL for Ms16 C121 and 2.5 µL for Ms33 Sol11 and Ms41 i134.

LITERATURE CITED

Tay WT, Crozier RH. 2000 Microsatellite analysis of gamergate relatedness of the queenless ponerine ant *Rhytidoponera sp. 12.* Insectes Soc. 47, 188–192. (doi:10.1007/PL00001700)

Table B1. Microsatellite primer sequences, types of simple sequence repeats (SSR), annealing temperatures, allele size range for *S. geminata*, and references from which the primer sequences were adapted.

Locus	Primer sequence	SSR units	Annealing temperature	Allele size range	Reference
Ms16	F:NED-CGTGGAGGGACGATTTTCATACTC	$(GA)_{15}$	58˚C	175-197	Wang et al. 2007
C121[1]	R[2]:GCTTCTCAAACTTCTCTTCGGTGACACTCT				
Ms19	F:VIC-CCCGAACAATGAGACATCCT	$(GA)_{7}$	56˚C	244-253	Wang et al. 2007
C367[1]	R:TTAAATCTTAGCGCCGACGA				
Ms33	F:6FAM-ACTGGAGCCTCCGAGACC	$(TC)_{15}$	56˚C	132-180	Chen et al. 2003
Sol11[1]	R:CACTCCGGAAGAGTAACTTTGC				
Ms14	F:PET-TTTCTTTCTTTCTGTCTCTTTCTCG	$(TC)_{14}$	54˚C	197-220	Wang et al. 2007
C334	R:ACGGAAGGCACGAATGAAGTC				
Ms4	F:6FAM-TGCGACAATGAATGAGAGTC	$(TC)_{15}$	48˚C	187-205	Chen et al. 2003
Sol55	R:TGCGAATATCCGGTCGAG				
Ms41	F:PET-CAGCTAAAAGTCAGGGACACG	$(GT)_{10}$	56˚C	196-219	Ascunce et al.
i134	R:AGACGCTTTATTATCTGGGACAC				2011

[1] Primers used for queens, larvae and sperm

[2] GCTTCT ‘pigtail’ sequence

LITERATURE CITED

Ascunce MS, Yang C-C, Oakey J, Calcaterra L, Wu W-J, Shih C-J, Goudet J, Ross KG, Shoemaker DD. 2011 Global invasion history of the fire ant *Solenopsis invicta*. Science 331, 1066–1068. (doi:10.1126/science.1198734)

Chen YP, Lu LY, Skow LC, Vinson SB. 2003 Relatedness among co-existing queens within polygyne colonies of a Texas population of the fire ant, *Solenopsis invicta*. Southwest. Entomol. 28, 27–36.

Wang J, Jemielity S, Uva P, Wurm Y, Gräff J, Keller L. 2007 An annotated cDNA library and microarray for large-scale gene-expression studies in the ant *Solenopsis invicta*. Genome Biol. 8, R9. (doi:10.1186/gb-2007-8-1-r9)

Appendix C. Figures on the colony founding experiment on the number of worker brood and adult worker per treatment, the number of worker brood and adult workers per queen, the number of DMP colonies executing diploid male larvae per treatment, and the weight distribution of newly mated queens.

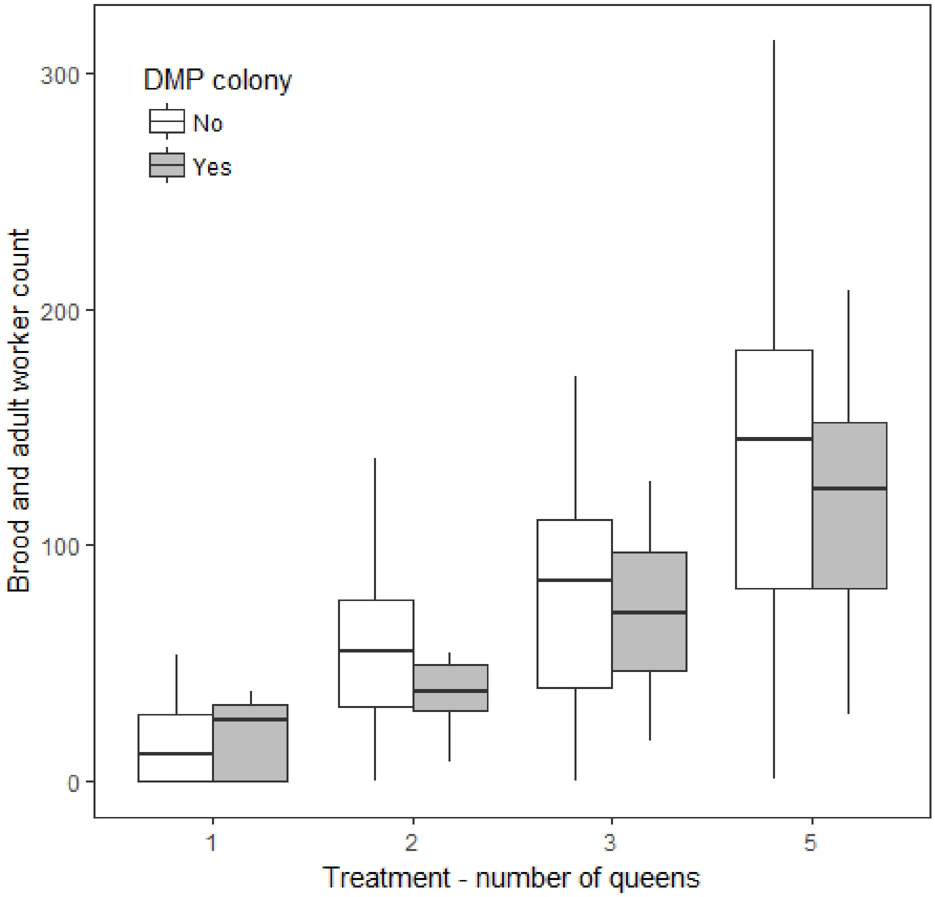

Figure C1. Number of worker brood and adult worker produced for each queen treatment separated between DMP (diploid male producing) colonies and non-DMP colonies. There was no significant difference between DMP and non-DMP colonies across all queen treatments (Table 1). One queen: non-DMP n = 97, DMP n = 5, two queens: non-DMP n = 79, DMP n = 16, three queens: non-DMP n = 68, DMP n = 29, five queens: non-DMP n = 51, DMP n = 35.

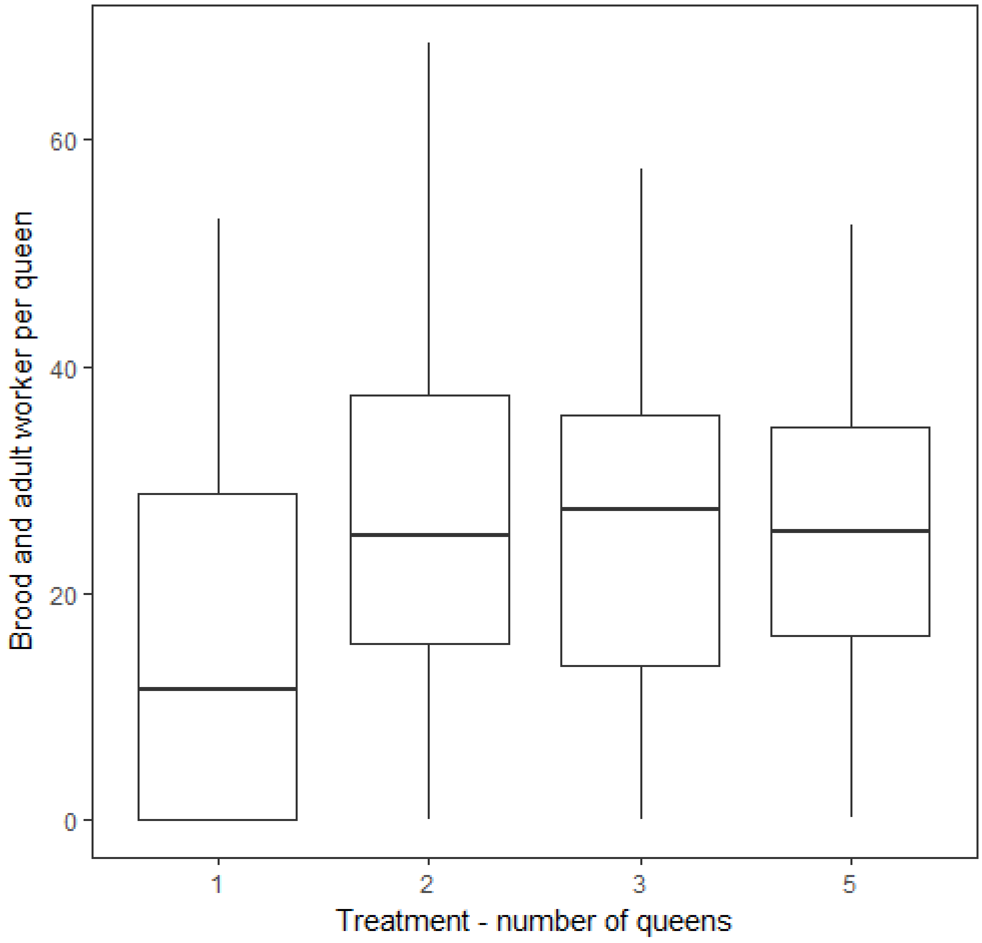

Figure C2. Number of brood and adult workers produced per queen (total number of brood and adult workers divided by the number of queens) for each queen treatment. The queen treatment had a significant effect on the number of worker brood and adult workers produced per queen but none of the pairwise comparisons between queen treatment were significant (LM: ANOVA, χ^2 = 36.038, P<0.01; post hoc tests P>0.05 for all pairwise comparisons). One queen: n = 102, two queens: n = 95, three queens: n = 97, five queens: n = 86.

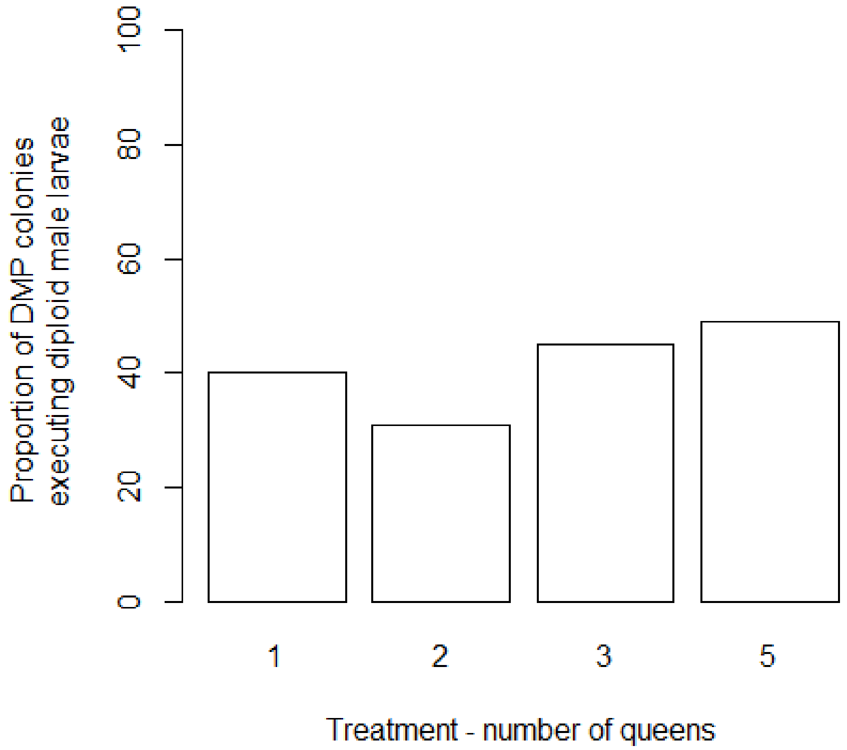

Figure C3. Proportion of DMP colonies executing diploid male larvae for each queen treatment. One queen: DMP n = 5, two queens: DMP n = 16, three queens: DMP n = 29, five queens: DMP n = 35.

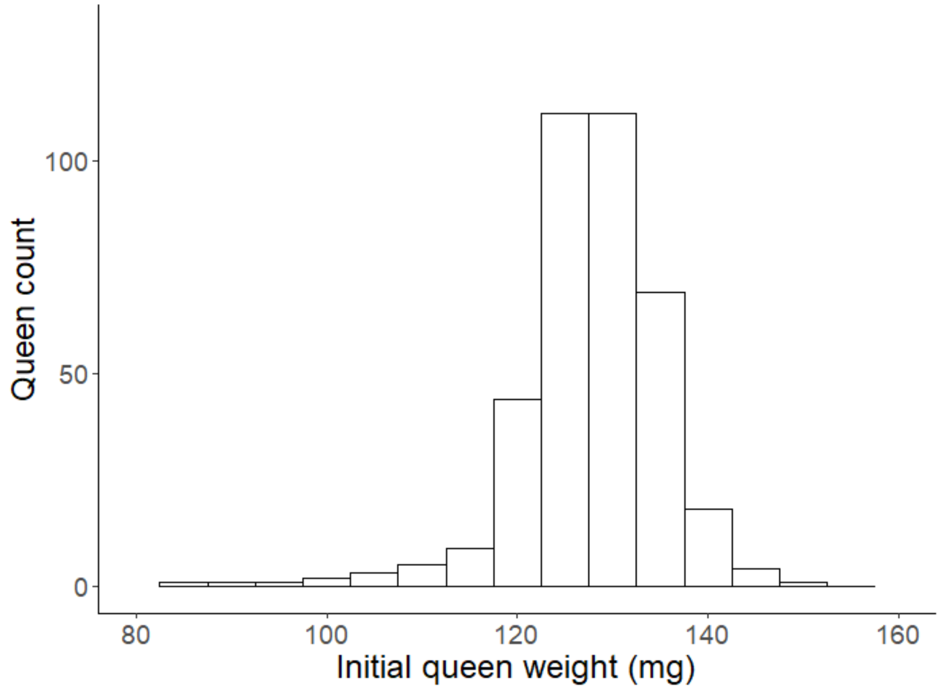

Figure C4. Weight distribution of the newly mated queens used in the colony founding experiment before the start of the experiment (n = 1,013).

Appendix D. Probability of double mating and queen and sperm genotypic distribution

We calculated the probability of not having detected double-mating in our population using the sperm genotype frequency from 32 males (Table D2) which were successfully genotyped at all three loci. The probability of homogeneous mating for each genotype was calculated by squaring the genotypic frequency. To obtain the probability of not detecting a single double-mating event in our sample, we summed the probability of homogeneous mating for each genotype which was 0.121. To obtain the probability of not detecting more than one double mating event we raised 0.121 to the power of the number of double mating events (Fig. D1). For example, the probability of not detecting that three queens were double mated (i.e. about 10% of double mating) is $0.121^3=1.8x10^{-3}$, ten double mated queens (i.e. about 30% double mating) $0.121^{10}=6.7x10^{-10}$ etc.

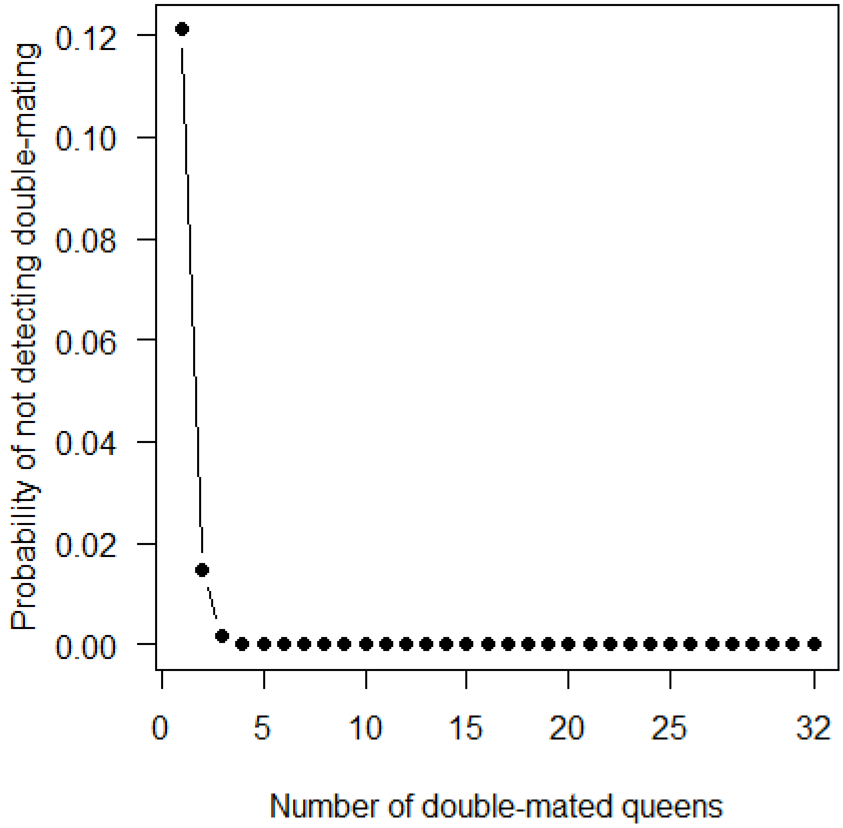

Figure D1. Probability of not detecting double-mating calculated from 32 sperm genotype frequencies

Table D1. Genotypic distribution of queens

ms33 Sol11		ms16 c121		ms41-i134		Sample size
132	132	190	190	202	202	1
134	134	190	190	202	220	1
134	134	190	190	202	221	3
134	134	190	190	221	221	1
134	134	192	192	220	220	1
134	163	188	188	220	220	1
134	179	190	190	202	202	1
163	163	190	190	221	221	1
163	179	190	190	202	220	1
177	177	190	190	202	221	1
179	179	192	192	202	220	1
134	177	190	190	221	221	2
129	129	190	190	-1	-1	1
179	179	190	190	-1	-1	1
163	179	190	190	-1	-1	1
134	179	192	192	-1	-1	1
134	177	190	190	-1	-1	2
135	135	-1	-1	202	202	1
177	177	-1	-1	202	202	1
-1	-1	190	190	202	202	1
179	179	-1	-1	-1	-1	1
134	134	-1	-1	-1	-1	5
134	179	-1	-1	-1	-1	2
-1	-1	190	190	-1	-1	1
-1	-1	-1	-1	202	220	2
-1	-1	-1	-1	202	221	1

Table D2. Genotypic distribution of sperm samples

ms33 Sol11	ms16 c121	ms41-i134	Sample size
134	190	202	8
134	190	220	1
134	190	221	1
134	192	202	6
134	192	221	2
134	194	221	1
163	190	221	1
163	192	202	1
177	190	202	1
177	190	221	1
177	190	223	1
177	192	221	1
179	186	202	1
179	190	202	2
179	192	202	2
179	192	220	1
179	192	221	1
-1	189	220	1
-1	190	202	1
-1	192	202	2
-1	192	221	1
134	190	-1	1
134	194	-1	1
135	194	-1	1
179	190	-1	1
